# Supplementary material for: Ovarian cancer prevention through opportunistic salpingectomy during abdominal surgeries: A cost-effectiveness modeling study
Source: PLoS Med. 2025 Jan 30;22(1):e1004514. doi: 10.1371/journal.pmed.1004514 (PMC11781718; doi:10.1371/journal.pmed.1004514)
Supplement: S1 Text — Table A: Surgeries with opportunity for OS included in each strategy. Table B: Background mortality for women in Germany 2018–2020. Table C: Age-dependent ovarian cancer mortality. Table D: Age-dependent risk (transition probability) for diagnosis of ovarian cancer. Table E: Age-dependent relative survival rates after ovarian cancer diagnosis. Table F: Risk of dying after ovarian cancer diagnosis (hazard = transition probability, P). Tables G and H: Age-dependent risk (transition probability) for gynecologic surgery with opportunity for OS. Table I: Age-dependent risk (transition probability) for non-gynecologic abdominal surgery with opportunity for OS. Table J: Recalculation of follow-up (FU) costs including maintenance therapy. Table K: Calculation of ovarian cancer primary therapy and follow-up (FU) costs. Fig A: Monte Carlo Microsimulation with increasing numbers of individuals until stable low variance was achieved. Fig B: Monte Carlo Microsimulation with increasing numbers of individuals until stable ovarian cancer rate was achieved. Fig C: Monte Carlo Microsimulation with increasing numbers of individuals until stable ICER was achieved. Table L: Results of one-way deterministic sensitivity analysis regarding clinical outcome. Table M: Results of deterministic sensitivity analysis regarding health-economic outcome. Table N: Characteristics of distributions used for probabilistic sensitivity analysis. Fig D: Proportion of simulations giving the indicated percentage of prevented ovarian cancer cases in probabilistic sensitivity analysis. (DOCX) [file pmed.1004514.s001.docx]

**Ovarian cancer prevention through opportunistic salpingectomy during abdominal surgeries: A cost-effectiveness modelling study**

*Angela Kather*^1,6^**, Habib Arefian*^2,4^*, Claus Schneider*^3^*, Michael Hartmann*^2^*, Ingo B. Runnebaum*^1,5,6^*

1Department of Gynecology and Reproductive Medicine, University Hospital Jena, Friedrich Schiller University Jena, Jena, Germany.

2Hospital Pharmacy, University Hospital Jena, Friedrich Schiller University Jena, Jena, Germany.

3Department of General, Visceral and Vascular Surgery, Jena University Hospital, Friedrich Schiller University

Jena, Jena, Germany.

4Present address: BIG direkt gesund, Berlin, Germany

5RU21 GmbH, Jena, Germany

6 Zentrum für Alternsforschung Jena – Aging Research Center Jena, Jena, Germany

*Corresponding author

Regarding model and analysis: Dr. rer. nat. Angela Kather, MSc

E-mail: [angela.kather@med.uni-jena.de](mailto:angela.kather@med.uni-jena.de)

ORCID ID: 0000-0001-7652-698X

Regarding surgery and gynecology: Univ.-Prof. Dr. med. Ingo B. Runnebaum, MBA
E-mail: [ingo.runnebaum@med.uni-jena.de](mailto:ingo.runnebaum@med.uni-jena.de)

Phone:  +49 3641 9329 133

**Supplementary Materials - Index**

| **Supplementary Figures and Tables** | *Page* |
| --- | --- |
| Table A | *2* |
| Table B | *3* |
| Tables C-D | *4* |
| Tables E-F | *5* |
| Tables G-H | *6* |
| Table I | *7* |
| Tables J-K | *8* |
| Figures A-B | *9* |
| Figure C | *10* |
| Tables L-M | *11* |
| Table N and Figure D | *12* |
| **References** | *13* |

**Supplementary Figures and Tables**

Supplementary Table A: **Surgeries with opportunity for OS included in each strategy.**

a, Inpatient case numbers of all German public hospitals for the year 2019 obtained from DESTATIS (1). b, Based on this number, transition probabilities for sterilization used in the model were calibrated to obtain the rate of 5% women in Germany, who claim to have undergone sterilization (2) c, Total case number for the respective strategy.

| **Strategy** | **Surgeries with opportunity for OS** | | | | |
| --- | --- | --- | --- | --- | --- |
|  | **Surgery** | | **OPS Code** | **Included cases** | **^a^Case number 2019** |
| I (Gyn+NonGyn) | Gyn | Benign hysterectomy without BSO | 5-682 +  5-683 | All | 72,163 |
|  |  | Sterilisation (inpatient) | 5-663 | ^b^All | ^b^10,156 |
|  |  | Sectio Caesarea | 5-740 +  5-741 +  5-742 +  5-749 | All > 40 years | 20,405 |
|  |  | Ovarian cyst removal | 5-651 | All > 40 years | 17,289 |
|  |  | Endometriosis surgery | ICD-10 N80 (inpatients) | 50% > 40 years | 5,503 |
|  |  | Myomectomy lapraroscopic or abdominal | 5-681 | All > 40 years | 5,383 |
|  |  | Uterus fixation lapraroscopic or abdominal | 5-704 | All > 40 years | 1,356 |
|  | NonGyn | Cholecystectomy | 5-511 | All > 40 years | 92,013 |
|  |  | Hernia closure (inguinalis, umbilicalis, incisional) | 5-530 +  5-534 +  5-536 | All > 40 years | 47,327 |
|  |  | Bariatric surgery | 5-434 +  5-435 +  5-445 | All > 40 years | 11,341 |
|  |  | Appendectomy | 5-470 | All > 40 years | 3,800 |
|  |  | | | | **^c^286,736** |
| II  (Gyn) | Gyn | Benign hysterectomy without BSO | 5-682 +  5-683 | All | 72,163 |
|  |  | Sterilisation (inpatient) | 5-663 | ^b^All | ^b^10,156 |
|  |  | Sectio Caesarea | 5-740 +  5-741 +  5-742 +  5-749 | All > 40 years | 20,405 |
|  |  | Ovarian cyst removal | 5-651 | All > 40 years | 17,289 |
|  |  | Endometriosis surgery | ICD-10 N80 (inpatients) | 50% > 40 years | 5,503 |
|  |  | Myomectomy lapraroscopic or abdominal | 5-681 | All > 40 years | 5,383 |
|  |  | Uterus fixation lapraroscopic or abdominal | 5-704 | All > 40 years | 1,356 |
|  | NonGyn | - | - | - | - |
|  |  | | | | **^c^132,255^c^** |
| III  (HE+Steri) | Gyn | Benign hysterectomy without BSO | 5-682 +  5-683 | All | 72,163 |
|  |  | Sterilisation (inpatient) | 5-663 | ^b^All | ^b^10,156 |
|  | NonGyn | - | - | - | - |
|  |  | | | | **^c^82,319** |
| IV  (No OS) | Gyn | - | - | - | - |
|  | NonGyn | - | - | - | - |

Supplementary Table B: **Background mortality** for women in Germany 2018-2020 obtained on 8/2/2023 from DESTATIS (1).

| **Age** | **Probability of dying (mortality table from DESTATIS)** | **Age** | **Probability of dying**  **(mortality table from DESTATIS)** |
| --- | --- | --- | --- |
| 20 | 0.00016368 | 68 | 0.01020397 |
| 21 | 0.00016980 | 69 | 0.01154629 |
| 22 | 0.00016778 | 70 | 0.01255177 |
| 23 | 0.00016356 | 71 | 0.01381449 |
| 24 | 0.00016427 | 72 | 0.01494790 |
| 25 | 0.00019441 | 73 | 0.01673605 |
| 26 | 0.00017824 | 74 | 0.01819212 |
| 27 | 0.00020975 | 75 | 0.02011999 |
| 28 | 0.00023799 | 76 | 0.02214882 |
| 29 | 0.00024676 | 77 | 0.02393844 |
| 30 | 0.00028279 | 78 | 0.02720749 |
| 31 | 0.00032200 | 79 | 0.03080839 |
| 32 | 0.00033591 | 80 | 0.03526151 |
| 33 | 0.00035023 | 81 | 0.04041529 |
| 34 | 0.00038179 | 82 | 0.04700229 |
| 35 | 0.00046694 | 83 | 0.05519587 |
| 36 | 0.00047686 | 84 | 0.06378801 |
| 37 | 0.00050493 | 85 | 0.07369375 |
| 38 | 0.00055411 | 86 | 0.08527851 |
| 39 | 0.00065065 |  |  |
| 40 | 0.00063313 |  |  |
| 41 | 0.00073294 |  |  |
| 42 | 0.00080685 |  |  |
| 43 | 0.00089569 |  |  |
| 44 | 0.00094585 |  |  |
| 45 | 0.00107566 |  |  |
| 46 | 0.00118419 |  |  |
| 47 | 0.00129937 |  |  |
| 48 | 0.00151711 |  |  |
| 49 | 0.00168407 |  |  |
| 50 | 0.00184390 |  |  |
| 51 | 0.00207651 |  |  |
| 52 | 0.00220442 |  |  |
| 53 | 0.00247858 |  |  |
| 54 | 0.00275427 |  |  |
| 55 | 0.00311238 |  |  |
| 56 | 0.00338159 |  |  |
| 57 | 0.00375629 |  |  |
| 58 | 0.00417358 |  |  |
| 59 | 0.00463446 |  |  |
| 60 | 0.00512681 |  |  |
| 61 | 0.00558618 |  |  |
| 62 | 0.00612191 |  |  |
| 63 | 0.00676019 |  |  |
| 64 | 0.00740880 |  |  |
| 65 | 0.00801369 |  |  |
| 66 | 0.00875776 |  |  |
| 67 | 0.00935793 |  |  |

Supplementary Table C**: Age-dependent ovarian cancer mortality.** Number of ovarian cancer deaths was obtained on 7/25/2023 from German Centre for Cancer Registry Data (3).

Number of women in Germany in each age group at the beginning of 2019 was obtained on 7/25/2023 from DESTATIS online database (1).

| **Age** | **Number of ovarian cancer deaths 2019 in each age group = I** | **Number of women at risk (Number of women in Germany in each age group at the beginning of 2019) = N_0_** | **Yearly risk of dying from ovarian cancer P = I/N_0_** |
| --- | --- | --- | --- |
| 20 - 24 | 1 | 2192260 | 4.5615E-07 |
| 25 - 29 | 11 | 2497399 | 4.40458E-06 |
| 30 - 34 | 14 | 2624948 | 5.33344E-06 |
| 35 – 39 | 26 | 2584099 | 1.00615E-05 |
| 40 - 44 | 48 | 2402780 | 1.99769E-05 |
| 45 – 49 | 103 | 2772302 | 3.71532E-05 |
| 50 - 54 | 257 | 3405726 | 7.54611E-05 |
| 55 - 59 | 409 | 3300544 | 0.000123919 |
| 60 - 64 | 483 | 2798189 | 0.000172612 |
| 65 - 69 | 618 | 2516863 | 0.000245544 |
| 70 - 74 | 583 | 1916659 | 0.000304175 |
| 75 - 79 | 962 | 2265773 | 0.000424579 |
| 80 - 84 | 959 | 1824473 | 0.000525631 |
| 85 and older | 816 | 1539616 | 0.000530002 |

Supplementary Table D**: Age-dependent Risk (Transition probability) for diagnosis of ovarian cancer.** Ovarian cancer case numbers were obtained on 03.07.2023 from German Centre for Cancer Registry Data (3). Number of women in Germany in each age group at the beginning of 2019 was obtained on 7/25/2023 from DESTATIS online database (1).

| **Age** | **Number of ovarian cancer cases 2019 in each age group = I** | **Number of women at risk (Number of women in Germany in each age group at the beginning of 2019) = N_0_** | **Yearly risk of ovarian cancer diagnosis P = I/N_0_** |
| --- | --- | --- | --- |
| 20 - 24 | 28 | 2192260 | 0.000012772 |
| 25 - 29 | 52 | 2497399 | 0.000020822 |
| 30 - 34 | 63 | 2624948 | 0.000024000 |
| 35 – 39 | 103 | 2584099 | 0.000039859 |
| 40 - 44 | 181 | 2402780 | 0.000075329 |
| 45 – 49 | 299 | 2772302 | 0.000107853 |
| 50 - 54 | 545 | 3405726 | 0.000160025 |
| 55 - 59 | 823 | 3300544 | 0.000249353 |
| 60 - 64 | 833 | 2798189 | 0.000297693 |
| 65 - 69 | 893 | 2516863 | 0.000354807 |
| 70 - 74 | 730 | 1916659 | 0.000380871 |
| 75 - 79 | 1105 | 2265773 | 0.000487692 |
| 80 - 84 | 918 | 1824473 | 0.000503159 |

Supplementary Table E**: Age-dependent relative survival rates after ovarian cancer diagnosis** (years 2017-2018) were obtained on 8/9/2023 from German Centre for Cancer Registry Data (3).

|  | Relative survival rate dependent on age [years] | | | | |
| --- | --- | --- | --- | --- | --- |
| Years after diagnosis | 15 - 44 | 45 - 54 | 55 - 64 | 65 - 74 | 75 and older |
| 0 | 1 | 1 | 1 | 1 | 1 |
| 1 | 0.95 | 0.91 | 0.86 | 0.79 | 0.49 |
| 2 | 0.91 | 0.81 | 0.74 | 0.67 | 0.37 |
| 3 | 0.85 | 0.74 | 0.64 | 0.55 | 0.28 |
| 4 | 0.82 | 0.69 | 0.56 | 0.45 | 0.23 |
| 5 | 0.77 | 0.64 | 0.48 | 0.39 | 0.21 |
| 6 | 0.75 | 0.6 | 0.44 | 0.33 | 0.19 |
| 7 | 0.74 | 0.57 | 0.42 | 0.3 | 0.18 |
| 8 | 0.72 | 0.53 | 0.39 | 0.28 | 0.18 |
| 9 | 0.71 | 0.52 | 0.38 | 0.26 | 0.18 |
| 10 | 0.71 | 0.51 | 0.36 | 0.26 | 0.19 |

Supplementary Table F: **Risk of dying after ovarian cancer diagnosis** (hazard = transition probability, *P*) was calculated from survival rates using the formula

 (*y* = survival rate from table above at time *t)*

According to (4).

|  | Risk of dying (Hazard) after ovarian cancer diagnosis dependent on age | | | | |
| --- | --- | --- | --- | --- | --- |
| Years after diagnosis | 15 - 44 | 45 - 54 | 55 - 64 | 65 - 74 | 75 and older |
| 1 | 0.050000000 | 0.0900000 | 0.1400000 | 0.2100000 | 0.5100000 |
| 2 | 0.046060799 | 0.1000000 | 0.1397675 | 0.1814647 | 0.3917237 |
| 3 | 0.052731763 | 0.0954958 | 0.1382261 | 0.1806787 | 0.3457867 |
| 4 | 0.048402126 | 0.0885932 | 0.1349385 | 0.1809637 | 0.3074806 |
| 5 | 0.05093022 | 0.0853899 | 0.1365280 | 0.1716518 | 0.2681133 |
| 6 | 0.046815707 | 0.0816141 | 0.1278816 | 0.1687104 | 0.2417852 |
| 7 | 0.042102991 | 0.0771630 | 0.1165571 | 0.1580176 | 0.2172729 |
| 8 | 0.040231345 | 0.0762925 | 0.1110382 | 0.1471066 | 0.1929340 |
| 9 | 0.037339505 | 0.0700817 | 0.1019319 | 0.1390121 | 0.1734816 |
| 10 | 0.033669172 | 0.0651175 | 0.0971195 | 0.1260284 | 0.1530157 |

Supplementary Table G**: Age-dependent risk (transition probability) for gynecologic surgery with opportunity for OS.** Age-dependent cases numbers were obtained via Email contact from the Federal Statistical Office of Germany (DESTATIS) (1).

| **Age in years** | **Number of women at risk (Number of women in Germany in each age group at the beginning of 2019) = N_0_** | **Benign hysterectomy without BSO** | | **Myomectomy lapraroscopic or abdominal** | | **Sterilisation (inpatient)** | |
| --- | --- | --- | --- | --- | --- | --- | --- |
|  |  | **Cases 2019 = I** | **Yearly Risk**  **P = I/N_0_** | **Cases 2019 = I** | **Risk** | **Cases 2019 = I** | **Yearly Risk**  **P = I/N_0_** |
| 20 - 24 | 2192260 | 31 | 1.41407E-05 |  |  | 116 | 5.29134E-05 |
| 25 - 29 | 2497399 | 380 | 0.000152158 |  |  | 1.041 | 0.000416834 |
| 30 - 34 | 2624948 | 2060 | 0.000784777 |  |  | 3.258 | 0.001241167 |
| 35 – 39 | 2584099 | 6390 | 0.002472815 |  |  | 3.873 | 0.001498782 |
| 40 - 44 | 2402780 | 13574 | 0.005649290 | 2375 | 0.000988438 | 1.538 | 0.000640092 |
| 45 – 49 | 2772302 | 18753 | 0.006764415 | 1294 | 0.000466760 | 228 | 8.22421E-05 |
| 50 - 54 | 3405726 | 12608 | 0.003702001 | 654 | 0.000192030 | 50 | 1.46812E-05 |
| 55 - 59 | 3300544 | 5176 | 0.001568226 | 383 | 0.000116041 | 19 | 5.75663E-06 |
| 60 - 64 | 2798189 | 3592 | 0.001283687 | 222 | 7.93370E-05 | 11 | 3.93111E-06 |
| 65 - 69 | 2516863 | 3233 | 0.001284536 | 187 | 7.42988E-05 | 8 | 3.17856E-06 |
| 70 - 74 | 1916659 | 2510 | 0.001309570 | 122 | 6.36524E-05 | 7 | 3.65219E-06 |
| 75 - 79 | 2265773 | 2508 | 0.001106907 | 93 | 4.10456E-05 | 6 | 2.64810E-06 |
| 80 - 84 | 1824473 | 1348 | 0.000738843 | 53 | 2.90495E-05 | 1 | 5.48103E-07 |

Supplementary Table H: **Age-dependent risk (transition probability) for gynecologic surgery with opportunity for OS.** Age-dependent cases numbers were obtained via Email contact from the Federal Statistical Office of Germany (DESTATIS) (1).

| **Age in years** | **Number of women at risk (Number of women in Germany in each age group at the beginning of 2019) = N_0_** | **Sectio caesarea** | | **Ovarian cyst removal** | | **Endometriosis surgery** | | **Uterus fixation lapraroscopic or abdominal** | |
| --- | --- | --- | --- | --- | --- | --- | --- | --- | --- |
|  |  | **Cases 2019 = I** | **Yearly Risk**  **P = I/N_0_** | **Cases 2019 = I** | **Yearly Risk**  **P = I/N_0_** | **Cases 2019 = I** | **Yearly Risk**  **P = I/N_0_** | **Cases 2019 = I** | **Yearly Risk**  **P = I/N_0_ (x10^-5^)** |
| 40 - 44 | 2402780 | 18921 | 0.00787463 | 7022 | 0.00292245 | 2391 | 0.0009951 | 88 | 3.6624 |
| 45 – 49 | 2772302 | 1314 | 0.00047397 | 5769 | 0.00208094 | 1803 | 0.00065018 | 109 | 3.9318 |
| 50 - 54 | 3405726 | 148 | 4.3456E-05 | 2738 | 0.00080394 | 875 | 0.00025692 | 168 | 4.9329 |
| 55 - 59 | 3300544 | 22 | 6.6656E-06 | 681 | 0.00020633 | 201 | 6.0899E-05 | 234 | 7.0897 |
| 60 - 64 | 2798189 | 0 | 0 | 318 | 0.00011364 | 95 | 3.3772E-05 | 192 | 6.8616 |
| 65 - 69 | 2516863 | 0 | 0 | 260 | 0.0001033 | 65 | 2.5627E-05 | 211 | 8.3835 |
| 70 - 74 | 1916659 | 0 | 0 | 177 | 9.2348E-05 | 43 | 2.2435E-05 | 171 | 8.9218 |
| 75 - 79 | 2265773 | 0 | 0 | 187 | 8.2533E-05 | 21 | 9.0477E-06 | 123 | 5.4286 |
| 80 - 84 | 1824473 | 0 | 0 | 137 | 7.509E-05 | 11 | 6.0291E-06 | 60 | 3.2886 |

Supplementary Table I**: Age-dependent risk (transition probability) for non-gynecologic abdominal surgery with opportunity for OS.** Age-dependent cases numbers were obtained via Email contact from the Federal Statistical Office of Germany (DESTATIS) (1).

| **Age in years** | **Number of women at risk (Number of women in Germany in each age group at the beginning of 2019) = N_0_** | **Cholecystectomy** | | **Hernia closure** | | **Bariatric surgery** | | **Appendectomy** | |
| --- | --- | --- | --- | --- | --- | --- | --- | --- | --- |
|  |  | **Cases 2019 = I** | **Yearly Risk**  **P = I/N_0_** | **Cases 2019 = I** | **Yearly Risk**  **P = I/N_0_** | **Cases 2019 = I** | **Yearly Risk**  **P = I/N_0_** | **Cases 2019 = I** | **Yearly Risk**  **P = I/N_0_** |
| 40 - 44 | 2402780 | 7954 | 0.00331033 | 3561 | 0.00148203 | 1972 | 0.00082072 | 2800 | 0.0011653 |
| 45 – 49 | 2772302 | 9329 | 0.00336507 | 4130 | 0.00148974 | 1951 | 0.00070375 | 2690 | 0.0009703 |
| 50 - 54 | 3405726 | 12137 | 0.00356370 | 5069 | 0.00148838 | 2148 | 0.0006307 | 3290 | 0.0009660 |
| 55 - 59 | 3300544 | 13126 | 0.00397692 | 5386 | 0.00163185 | 1758 | 0.00053264 | 3033 | 0.0009189 |
| 60 - 64 | 2798189 | 12023 | 0.00429671 | 5352 | 0.00191267 | 1131 | 0.00040419 | 2263 | 0.0008087 |
| 65 - 69 | 2516863 | 11494 | 0.00456680 | 5688 | 0.00225996 | 743 | 0.00029521 | 1792 | 0.000712 |
| 70 - 74 | 1916659 | 8580 | 0.00447654 | 5314 | 0.00277253 | 455 | 0.00023739 | 1195 | 0.0006235 |
| 75 - 79 | 2265773 | 9605 | 0.00423917 | 6992 | 0.00308592 | 634 | 0.00027982 | 1113 | 0.0004912 |
| 80 - 84 | 1824473 | 7765 | 0.00425602 | 5835 | 0.00319818 | 549 | 0.00030091 | 826 | 0.0004527 |

Supplementary Table J: **Recalculation of follow up (FU) costs including maintenance therapy** according to German treatment guidelines (5) and pharmacy retail price 2023.

| **Maintenance therapy regimen** | **Costs per one outpatient administration (without costs for carrier solution)** | **Costs for one 21-day treatment cycle (outpatient, without costs for carrier solution)** | **costs per day** | **Total costs for 2-year therapy (730 days)** | **Total costs per year distributed over 5 years FU** |
| --- | --- | --- | --- | --- | --- |
| Bevacizumab 15mg/kg BW (1200mg) 3-wkl. | €2,079.01 | €2,079.01 | €99.00 | €72,270.35 |  |
| Olaparib 300mg p.o. 2xdaily (600mg); 4x150mg | €205.35 | €4,312.35 | €205.35 | €149,905.50 |  |
| Sum | €2,284.36 | €6,391.36 | €304.35 | €222,175.85 | €44,435.17 |

Supplementary Table K: **Calculation of ovarian cancer primary therapy and follow up (FU) costs** according to proportion of early and advanced stages, as well as with costs for maintenance therapy included.

|  | **Early ovarian cancer costs** | **Early OC costs adjusted for proportion* (23%)** | **Advanced ovarian cancer costs** | **Advanced OC costs adjusted for proportion* (77%)** | **Sum** |
| --- | --- | --- | --- | --- | --- |
| Primary therapy costs according to (6) | €13,666.00 | €3,143.18 | €35,052.00 | €26,990.04 | €30,133.22 |
| Yearly FU costs over 5 years according (6) | €1,356.00 | €311.88 | €2,389.00 | €1,839.53 | €2,151.41 |
| Yearly FU costs over 5 years according (6) plus costs for maintenance therapy (see table S10) | €1,356.00 | €311.88 | €46,824.17 | €36,054.61 | €36,366.49 |

*Proportion of early and advanced stages according to (7).

Supplementary Figure A: **Monte Carlo Microsimulation with increasing numbers of individuals** until stable low variance was achieved.


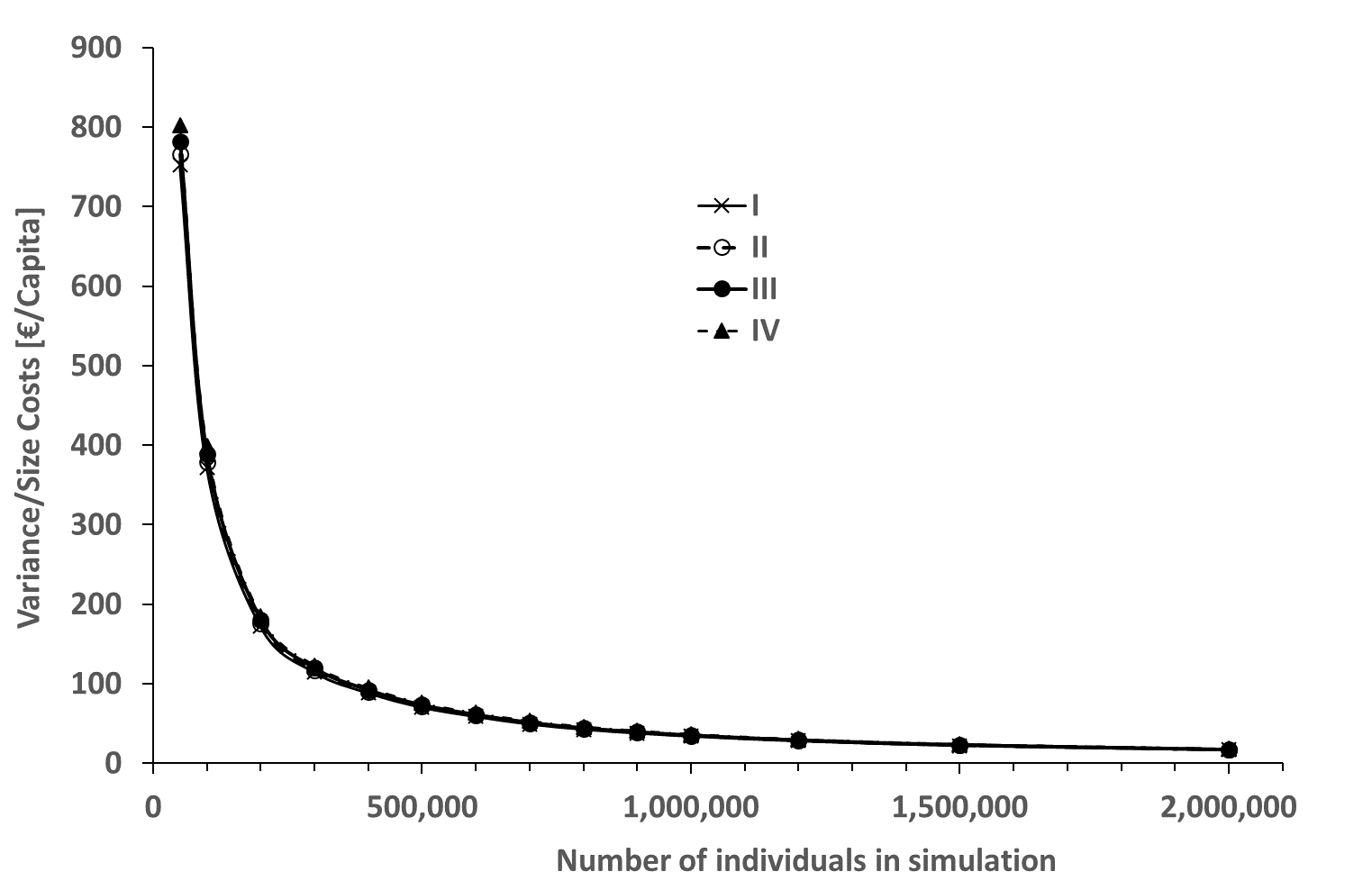


Supplementary Figure B: **Monte Carlo Microsimulation with increasing numbers of individuals** until stable ovarian cancer rate was achieved.


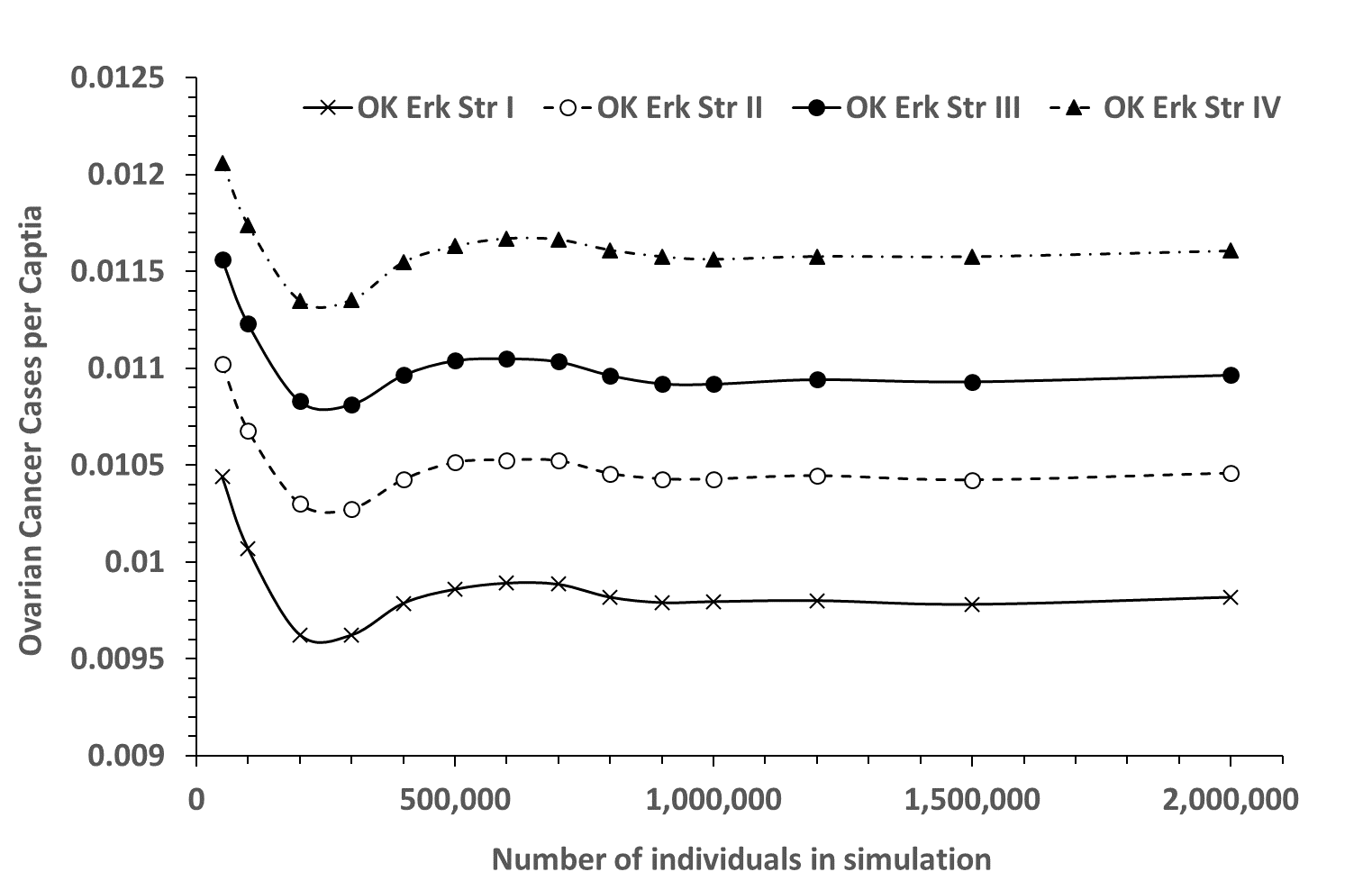


Supplementary Figure C: **Monte Carlo Microsimulation with increasing numbers of individuals** until stable ICER was achieved.


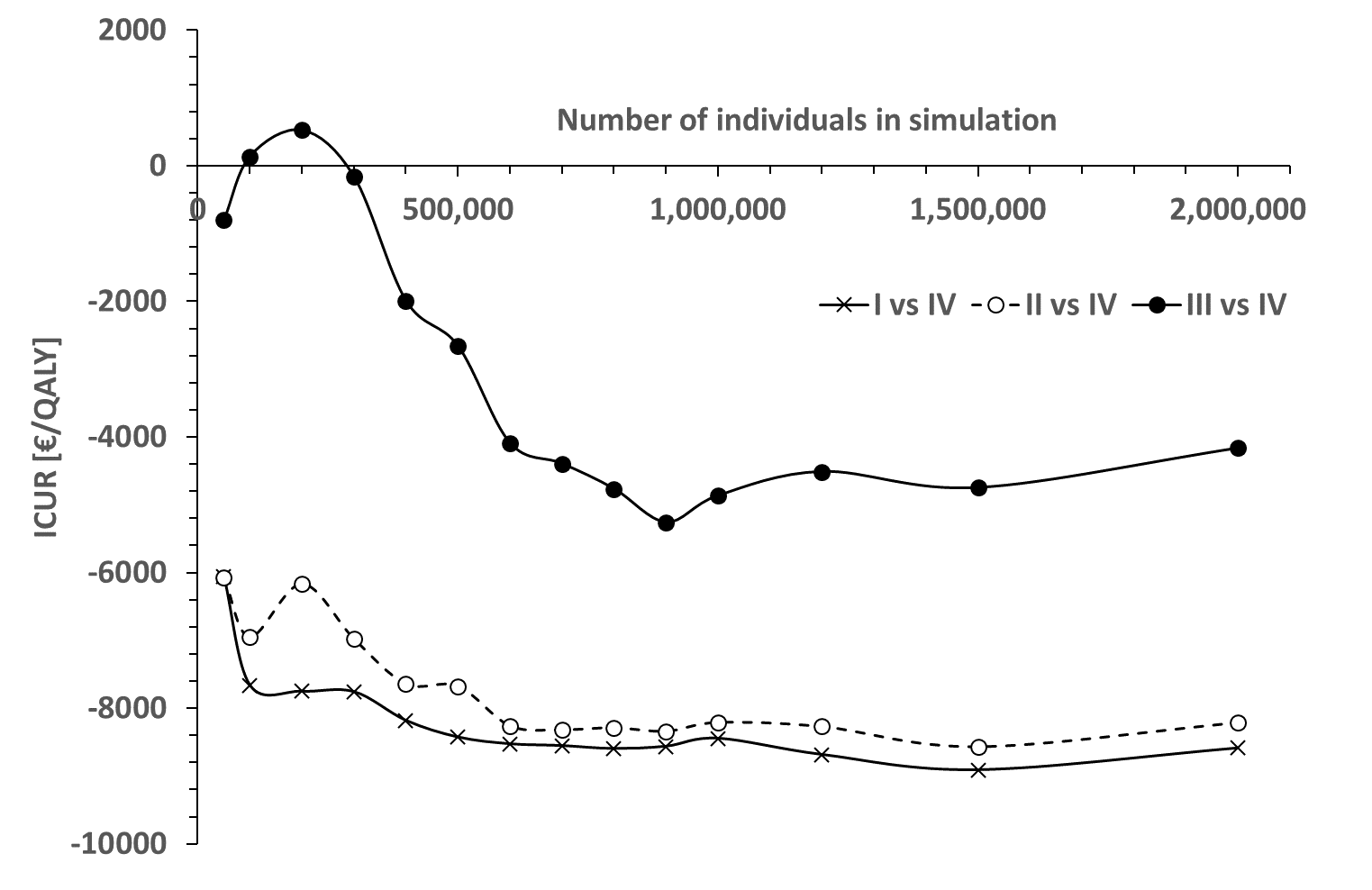


Supplementary Table L: **Results of one-way deterministic sensitivity analysis regarding clinical outcome:** prevented ovarian cancer cases compared to strategy IV (No OS)

| **Simulation cohort 1,200,000** | | **Strategy I vs. IV** | **Strategy II vs. IV** | **Strategy III vs. IV** |
| --- | --- | --- | --- | --- |
| **Ovarian cancer risk after salpingectomy (HR)** | 0.17 | 2824 (20.33%) | 1833 (13.19%) | 1088 (7.83%) |
|  | 0.35 (base case) | 2131 (15.34%) | 1359 (9.78%) | 762 (5.48%) |
|  | 0.45 | 1732 (12.47%) | 1070 (7.70%) | 566 (4.07%) |
|  | 0.54 | 1351 (9.72%) | 807 (5.81%) | 387 (2.79%) |
|  | 0.6 | 1099 (7.91%) | 629 (4.53%) | 286 (2.06%) |
|  | 0.73 | 565 (4.07%) | 264 (1.90%) | 51 (0.37%) |
| **Latency period (years)** | 0 | 2473 (17.80%) | 1503 (10.82%) | 865 (6.23%) |
|  | 5 (base case) | 2131 (15.34%) | 1359 (9.78%) | 762 (5.48%) |
|  | 10 | 1759 (12.66%) | 1180 (8.49%) | 628 (4.52%) |
|  | 15 | 1386 (9.98%) | 952 (6.85%) | 491 (3.53%) |
|  | 20 | 1005 (7.23%) | 726 (5.23%) | 350 (2.52%) |
| **Transition probability for hysterectomy** | 0.5x | 1972 (14.04%) | 1128 (8.03%) | 501 (3.57%) |
|  | 1x (base case) | 2131 (15.34%) | 1359 (9.78%) | 762 (5.48%) |
|  | 1.5x | 2315 (16.80%) | 1566 (11.36%) | 1022 (7.41%) |
| **Transition probability for sterilization** | 1x | 2041 (14.47%) | 1242 (8.86%) | 632 (4.51%) |
|  | 2.5x (base case) | 2131 (15.34%) | 1359 (9.78%) | 762 (5.48%) |
|  | 5x | 2255 (16.44%) | 1539 (11.22%) | 963 (7.02%) |
|  | 7.5x | 2404 (17.72%) | 1722 (12.70%) | 1183 (8.72%) |

Supplementary Table M: **Results of deterministic sensitivity analysis regarding health-economic outcome:** Incremental cost utility ratio (ICER, €/QALY) compared to strategy IV (No OS).

| **Simulation cohort 1,200,000** | | **Strategy I vs. IV** | **Strategy II vs. IV** | **Strategy III vs. IV** |
| --- | --- | --- | --- | --- |
| **Utility ovarian cancer** | 0.5 | -8237.32 | -7854.71 | -4287.00 |
|  | 0.61 (base case) | -8685.50 | -8270.55 | -4511.86 |
|  | 0.715 | -9161.29 | -8710.74 | -4749.67 |
|  | 0.82 | -9692.23 | -9200.43 | -5013.94 |
| **Ovarian cancer primary treatment costs (€)** | 15066.61 | -6042.56 | -5806.59 | -2073.55 |
|  | 22599.91 | -7364.03 | -7038.57 | -3292.70 |
|  | 30133.22 (base case) | -8685.50 | -8270.55 | -4511.86 |
|  | 45199.83 | -11328.43 | -10734.51 | -6950.18 |
|  | 60266.44 | -13971.37 | -13198.47 | -9388.50 |
| **Transition probability for hysterectomy** | 0.5x | -9170.37 | -8398.01 | -3906.87 |
|  | 1x (base case) | -8685.50 | -8270.55 | -4511.86 |
|  | 1.5x | -8618.09 | -7852.89 | -4997.19 |
| **Transition probability for sterilization** | 1x | -9384.42 | -9153.84 | -5293.67 |
|  | 2.5x (base case) | -868550 | -8270.55 | -4511.86 |
|  | 5x | -7837.30 | -7448.24 | -3989.34 |
|  | 7.5x | -7476.96 | -6953.86 | -4304.26 |
| **Discount rate** | 0 | -13794.13 | -13887.69 | -12361.35 |
|  | 0.03 (base case) | -8685.50 | -8270.55 | -4511.86 |
|  | 0.05 | 38.44 | 1698.08 | 9108.23 |
| **Ovarian cancer mortality** | 0.7 | -12942.11 | -12030.96 | -7684.44 |
|  | 1x (base case) | -8685.50 | -8270.55 | -4511.86 |

Supplementary Table N: **Characteristics of distributions used for probabilistic sensitivity analysis.**

|  | **Ovarian cancer risk reduction after salpingectomy** | **Costs of opportunistic salpingectomy** |
| --- | --- | --- |
| **Distribution** | LogNormal | LogNormal |
| **Median** | 0.35 | 214.96 |
| **95% CI** | 0.17 - 0.72 | 110.73 – 425.42 |
| **Mean** | 0.37 | 228.20 |
| **Min, Max** | 0.09, 1.30 | 60.73, 747.71 |

Supplementary Figure D: **Proportion of Simulations giving the indicated percentage of prevented ovarian cancer cases in probabilistic sensitivity analysis with the distributions described in S17**


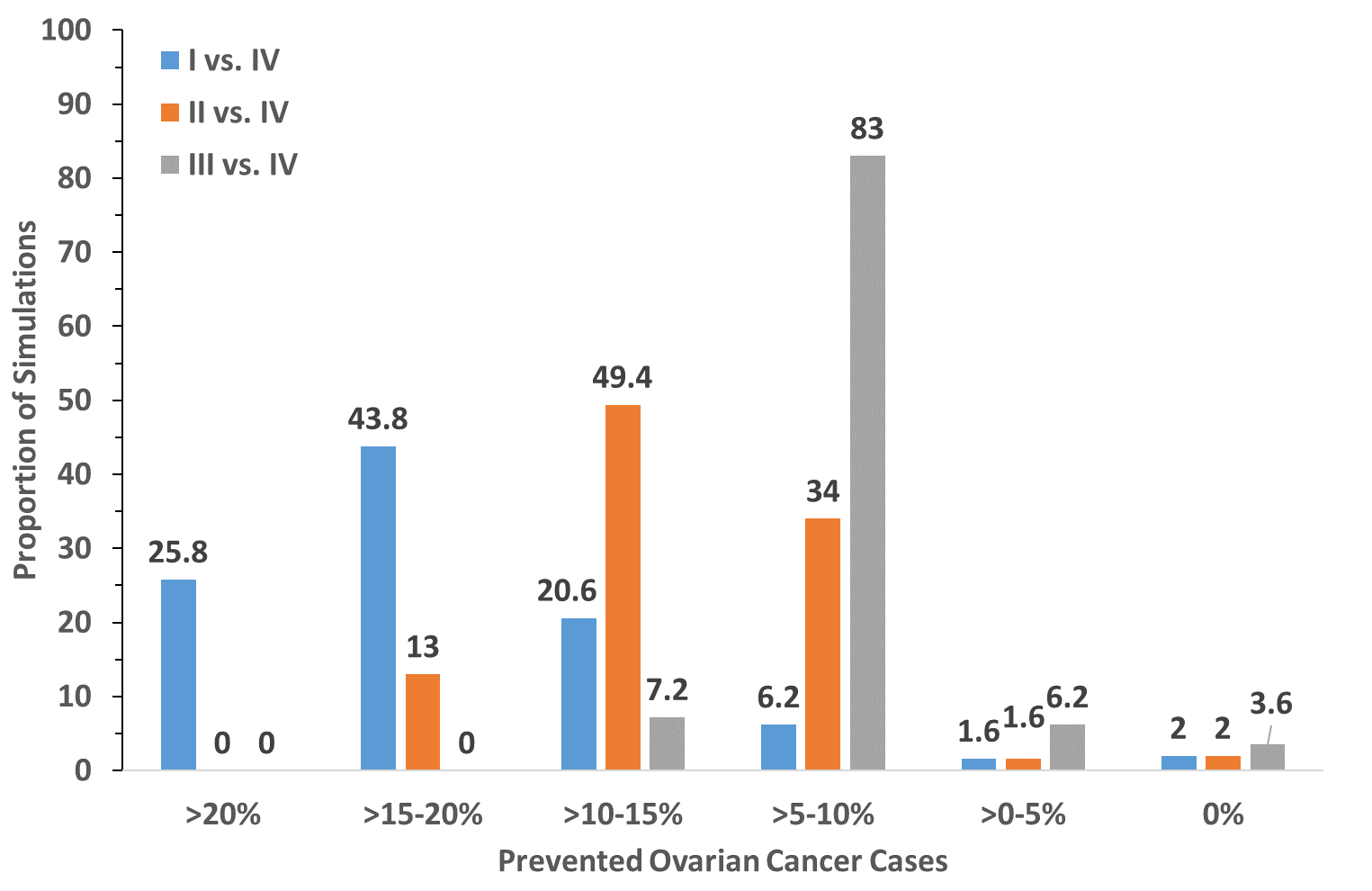


**References**

(1) Federal Statistical Office of Germany. Database:

<https://www-genesis.destatis.de/genesis/online>

Accessed on 3/19/2024

(2) Federal Centre for Health Education (2018): Contraceptive behaviour of adults. Results of the representative survey in Germany 2018. Available online at

<https://shop.bzga.de/verhuetungsverhalten-erwachsener-2018-13317300/>

Accessed on 9/28/2023

(3) German Centre for Cancer Registry Data. Database: <https://www.krebsdaten.de/Krebs/DE/Datenbankabfrage/datenbankabfrage_stufe1_node.html>

Accessed on 7/25/2023

(4) Sonnenberg FA. Beck JR. “Markov models in medical decision making: a practical guide. Medical Decision Making.” 1993; 13(4):322–38. Epub 1993/10/01. <https://doi.org/10.1177/0272989X9301300409> PMID: 8246705.

(5) German Guideline Program in Oncology. S3-Guideline on Diagnostics, Therapy and Follow-up of Malignant Ovarian Tumours - Long version: Version 5.1 2022.

<https://www.leitlinienprogramm-onkologie.de/fileadmin/user_upload/LL_Ovarialkarzinom_Langversion_5.1.pdf>

Accessed on 12/16/2022.

(6) Hallsson, Lára R.; Sroczynski, Gaby; Engel, Jutta; Siebert, Uwe (2023): Decision-analytic evaluation of the comparative effectiveness and cost-effectiveness of strategies to prevent breast and ovarian cancer in German women with BRCA-1/2 mutations. In BMC cancer 23 (1), p. 590. DOI: 10.1186/s12885-023-10956-6.

(7) Buttmann-Schweiger, Nina; Kraywinkel, Klaus (2019): Epidemiologie von Eierstockkrebs in Deutschland. In *Onkologe* 25 (2), pp. 92–98. DOI: 10.1007/s00761-018-0507-8.
