# Supplementary material for: Ovarian cancer prevention through opportunistic salpingectomy during abdominal surgeries: A cost-effectiveness modeling study
Source: PLoS Med. 2025 Jan 30;22(1):e1004514. doi: 10.1371/journal.pmed.1004514 (PMC11781718; doi:10.1371/journal.pmed.1004514)
Supplement: Attachment — Submitted filename: Point-by-point response letter final.docx [file pmed.1004514.s002.docx]

**Ovarian Cancer Prevention with Cost-Effective Opportunistic Salpingectomy—A Decision Analytic Assessment Across Abdominal Surgeries**

*Angela Kather, Habib Arefian, Claus Schneider, Michael Hartmann, Ingo B. Runnebaum*

PMEDICINE-D-24-02003R1

**Point-by-point Response to Editor and Reviewers**

Dear Dr. Tosun,

In the following sections, we provide a point-by-point response to each comment from the editor, academic editor, and reviewers, indicating where changes have been made in the manuscript (with page and line numbers where applicable).

**Section 1: Response to Academic Editor’s Comments**

**Comment 1 from the academic editor:**
“The comments of reviewers 1 and 2 are critical: in particular, histotype specificity for interventions (only high-grade serous carcinoma is thought to arise from the fallopian tube, other histotypes arise from different tissues and the risk reduction value is not known), … .”

**Authors’ response:**

To clarify the issue of varying risk reduction across different histologic subtypes, we have added a new paragraph titled “Basic Considerations and Assumptions” at the beginning of the Methods section (p. 7 lines 152-160). The paragraph reads as follows:

“Ovarian cancer risk reduction following the removal of fallopian tubes may vary by histologic subtype due to the diverse origins of precursor lesions. Additionally, some women, such as carriers of BRCA (BReast CAncer gene) mutations, have an increased risk of ovarian cancer. However, all data used to inform the model do not distinguish between histologic subtypes or between women with varying cancer risks. Consequently, the model’s results represent estimates of the impact of OS on overall ovarian cancer incidence, mortality, and healthcare costs at the population level. Women with an increased cancer risk require specialized consultation and are recommended to undergo bilateral salpingo-oophorectomy between the ages of 35 and 40.”

The confusing sentence in the limitations section has been removed (p. 19, line 486).

**“… number inflation.”**

**Authors’ response**:
The adjustment and alignment with the observed rate were necessary because most tubal sterilization procedures are performed in an outpatient setting and are not covered by the health insurance. Case numbers for such outpatient self-payed surgeries are currently not recorded in Germany. This is now explicitly stated on p. 9, line 212-219). In order to explore the impact of the used calibration and varying information regarding the observed rate on the results, different transition probabilities for sterilization were examined during sensitivity analysis (methods section p. 11, lines 284-287; results section p14 line 347; supplementary tables S15 and S16).

**The editorial team**
would like to note that, in line with the comments of reviewer 1, we would prefer that you reduce the number of abbreviations used throughout the manuscript.

**Authors’ response:**

We have decided to limit the use of abbreviations to figures and tables, and to write out terms in the main body of the manuscript. Exceptions include HR for hazard ratio, LY for life year, QALY for quality-adjusted life year and ICER for incremental cost-effectiveness ratio as these are well-known terms frequently used throughout the manuscript. The manuscript has been revised accordingly.

We also wondered if you could be more explicit about the absolute risk of ovarian cancer in the relevant group to contextualize "reduced by 15.3%" and the like.

**Authors’ response:**

Thank you for this important comment. We have now added the absolute numbers of ovarian cancer cases and deaths from the simulation cohort to Table 1, as well as the projected number of annual cases and deaths that could be prevented in Germany. This information has also been included in the main text on p.12 lines 315-317, to provide clearer context for the relative reduction figures.

**Section 2: Response to Reviewer #1’s Comments**

**Reviewer #1:** Thank you for allowing me to review this interesting manuscript. My comments are below.

Abstract

Line 28 - In general, the language here should be softened. We assume that the fallopian tubes are involved in ovarian cancer pathogenesis and removal reduces cancer risk based on observational data, but we do not know this for certain. This needs to be rephrased.

Line 44 - Again we do not know that we can prevent ovarian cancer by removing tubes, only reducing risk. please rephrase.

**Authors’ response:**

Thank you for your insightful feedback. We fully agree with this more cautious perspective on the current state of the literature. Accordingly, we have rephrased the relevant sentences on p. 2, lines 30-31, and lines 49-50 to reflect this view.

Introduction

Line 61 - Here hysterectomy and tubal ligation are abbreviated, and then these abbreviations are essentially never used again in the manuscript. Either use these abbreviations consistently, or do not define them at all.

**Authors’ response:**

Thank you for this observation. We have decided to limit the use of abbreviations to figures and tables, while writing out terms in the main body of the manuscript. Exceptions include HR for hazard ratio, LY for life year, QALY for quality-adjusted life year and ICER for incremental cost-effectiveness ratio as these are standard abbreviations frequently used throughout the manuscript. The manuscript has been revised accordingly.

Methods

Line 104 - during strategy 4, are the reference surgeries still performed, but with salpingectomy? or are no surgeries performed at all. this needs to be stated.

**Authors’ response:**

Thank you for highlighting this point. We assume that surgeries with the opportunity for OS are performed at the same frequency across all four strategies analyzed. Currently, it is not recommended to perform a surgery solely for the purpose of salpingectomy when a woman from the general population (i.e., non-BRCA carriers) desires a bilateral salpingectomy for risk reduction. Instead, OS is intended to be performed opportunistically during a medically necessary gynecological or non-gynecological abdominal intervention, or if a woman elects permanent contraception. To clarify this, we have added a new subsection titled “Basic Considerations and Assumptions” at the beginning of the Methods section (p. 7 lines 145-151).

Line 118 - is the model outlined in a figure? if not this would be helpful to understand how this was set up for the reader as this block of text is challenging to read without a visual. I think this is what is being conveyed in Figure 1 and this should be reference here if so.

**Authors’ response:**

*We now re-designed Figure 1 to enhance comprehensibility and reference figure 1 in the pertaining paragraph of the main text body (pp. 7-8 lines 164-195).*

Line 140 - why not use the raw numbers obtained from the inpatient data? why inflate it to match the rate observed in Germany?

**Authors’ response:**

The adjustment and alignment with the observed rate were necessary because most tubal sterilization procedures are performed in an outpatient setting and are not covered by the health insurance. Case numbers for such outpatient self-payed surgeries are currently not recorded in Germany. This is now explicitly stated on p. 9, lines 212-219. In order to explore the impact of the used calibration and varying information regarding the observed rate on the results, different transition probabilities for sterilization were examined during sensitivity analysis (methods section p. 11, lines 284-287; results section p 14 line 347; supplementary tables S15 and S16).

Line 173 - WTP presumably means willingness to pay but is never defined in the text

**Authors’ response:**

*Indeed, WTP was supposed to mean willingness-to-pay. However, we now decided to discuss our results in relation to the gross domestic product per capita, because no official willingness-to-pay threshold is defined for Germany.*

Line 187 - what is the difference between ICER and ICUR? This needs to be defined. Additionally, no primary outcome is ever stated, typically in cost effectiveness research, this is the ICER - is this what was considered the primary outcome for this manuscript?

**Authors’ response:**

*Sometimes, the ICER (incremental cost-effectiveness ratio) is called ICUR (cost-utility ratio) if it refers to costs per QALY gained (calculated based on utility of a life year in a specific condition) instead of costs per life year gained. In order to avoid confusion, we now defined our primary outcome to be the cost-effectiveness ratio (ICER) expressed as costs per QALY throughout the manuscript.*

Results

Line 226 - This paragraph should not be in the results section and instead should be moved to discussion.

**Authors’ response:**

*The paragraph was removed from the results section. Because most of the content was already present in the discussion, only one sentence was introduced on p. 16 lines 404-405.*

Line 238 - again the distinction between ICER and ICUR needs to be made to make sense of what this data is conveying.

**Authors’ response:**

*The paragraph on cost effectiveness in the results section (p. 14 lines 336-341) was re-structured and amended in order to make it more understandable.*

Discussion

Line 287 - In general I would reframe this pragraph to discuss how it is impractical to conduct randomized prospective research evaluating an outcome with such a small incidence as developing ovarian cancer. It is ethical to randomize patients to different treatment or prevention strategies when there is clinical equipoise as is the case in this situation, so I would remove that argument from this paragraph.

**Authors’ response:**

*We removed the sentence regarding ethical concerns from the paragraph.*

Line 323 "OS would be cost effective even if no maintenance therapy for ovarian cancer was applied" what does this mean? Was maintenance therapy factored into the model? This is not mentioned anywhere else. Are you referring to chemoprophylaxis with combined oral contraception? or maintenance medications for ovarian cancer like PARP inhibitors or avastin? Please clarify.

**Authors’ response:**

*To clarify this issue, we inserted “with PARP inhibitors and Bevacizumab” in the methods section (p. 10 line 246) and restructured and amended the paragraph in the discussion (p. 18 lines 456-463), which is now: “Model results were stable over a wide range of input parameters. OS would be cost-effective even if no maintenance therapy for ovarian cancer was applied. Since few years, PARP inhibitors are administered to patients with advanced ovarian cancer for up to two years after primary chemotherapy, sometimes in combination with Bevacizumab. This results in profoundly increased costs of more than 100,000 Euro per year of treatment (Supplemental table S10). Importantly, the degree of ovarian cancer risk reduction after OS appeared to be a crucial factor for effectiveness….”*

Line 339 - the emphasis here needs to be placed on the fact that this is a model and due to its inputs, it can only predict and therefore definitive conclusions cannot be made from this data. Because all of this data regarding risk reduction is based on knowledge from serous histologies, you really cannot say there are differences in risk reduction based on different histologies, because we have no data in the model to give you a sense of how this would affect someone with a clear cell carcinoma risk for example. I would remove this and make it clear this cannot be applied to a non serous setting, and furthermore, cannot be applied to patients without average background risk of ovarian cancer (ie. patients with BRCA mutations or at elevated risk of ovarian cancer due to family history).

**Authors’ response:**

*We included the sentence “However, definitive conclusions should not be drawn solely based on results of this model” in the limitations section on p. 18 lines 478-479.*

*In order make the issues of different risk reduction for histologic subtypes and higher cancer risk in women with BRCA mutations more clear, we included the following statement into a new paragraph “Basic considerations and assumptions” at the beginning of the methods section: “Ovarian cancer risk reduction after removal of fallopian tubes might be different for histologic subtypes, due to the divers origin of precursor lesions. Furthermore, there are women with increased ovarian cancer risk, e.g. BRCA (BReast CAncer gene) mutation carriers. However, all numbers used to inform the model do not discriminate between histological subtypes and between women with different cancer risk. Consequently, the results of the model represent estimates for the impact of OS on overall ovarian cancer incidence, mortality and healthcare costs on a population level. Women with increased cancer risk need special consultation and are recommended to have bilateral salpingo-oophorectomy between 35 and 45 years of age.” (p. 7 lines 152-160). The confusing sentence in the limitations section has been removed (p. 19 line 486).*

**Section 3: Response to Reviewer #2’s Comments**

**Reviewer #2:** Introduction. Key terminology missing here in this section is STIC (serous tubal intraepithelial carcinoma).

**Authors’ response:**

*We inserted the sentence “For serous tubal intraepithelial carcinomas (STICs) it was shown that they carry the same tumor-specific genetic alterations as the concomitant ovarian cancer lesions.” (p. 5, lines 111-113)*

Ovarian cancer parameter (Table S1-6). One-size-fits-all type concept was used for ovarian cancer statistics / parameters. Based on histology types, stage distribution and prognosis are signifantly different. Also, salpingectomy is suggested to prevent certain epithelial type ovarian cancer. Ovarian cancer statistics for non-epithelial type (germ cell and sex cord-stromal) would not apply here and needed to be distinguished.

**Authors’ response:**

*In order make the issue of different risk reduction for histologic subtypes more clear, we included the following statement into a new paragraph “Basic considerations and assumptions” at the beginning of the methods section: “Ovarian cancer risk reduction after removal of fallopian tubes might be different for histologic subtypes, due to the divers origin of precursor lesions. Furthermore, there are women with increased ovarian cancer risk, e.g. BRCA (BReast CAncer gene) mutation carriers. However, all numbers used to inform the model do not discriminate between histological subtypes and between women with different cancer risk. Consequently, the results of the model represent estimates for the impact of OS on overall ovarian cancer incidence, mortality and healthcare costs on a population level. Women with increased cancer risk need special consultation and are recommended to have bilateral salpingo-oophorectomy between 35 and 45 years of age.” (p. 7 lines 152-160). The confusing sentence in the limitations section was removed (p. 19 line 486).*

Cost effective threshold. This may be asssesed for various threshold cutpoints. Ovarian cancer treatment has evolved significantly in the recent years. More modern and effective treatments via biological targeted therapy is significantly expensive compared to systemic chemotherapy alone as shown in Table S10. These including anti-angiogenc therapy as well as synthetic lethalty-targeting poly [ADP]-ribose inhibitor matintenance therapy. In addition, universal genetic testing for individiual with epithelial ovarian cancer diagnosis as well as companion diagnostic testing became standard care in the management, and consideration of this testing cost also needed to be accounted in the threshold evaluation.

**Authors’ response:**

*Testing was included in primary OC treatment costs^1^ and pricy maintenance therapy was included in follow up costs (supplemental tables S10-S11). Impact of variation of OC treatment costs was analysed in deterministic sensitivity analysis over a wide range (fig. 3D, p. 10 lines 242-249, p. 15 lines 368-369).*

*In order to enable consideration of different threshold cutpoints, 1x and 2x gross domestic product per capita in Germany (2022)^2^ was included in figure 3.*

Cesarean delivery. This was only described in supplemental table. As salpingectomy is frequently performed at cesarean delivery, clarification of non-gyn surg in the main text would be useful otherwise readers have to look supplemental file to find the word cesarean.

**Authors’ response:**

*Caesarean section is explicitly mentioned in the methods section on page 7 line 173 as well as in the legend of figure 2.*

**Section 4: Response to Reviewer #3’s Comments**

**Reviewer #3:** Thank you for allowing me to review this relevant state transition analysis. The paper is well written and concise. Based on the results of this study, health policy maker will have to consider the implementation and re-imbursement of opportunistic salpingectomy (OS) as an add-on procedure in all abdominal surgical interventions. Congratulations.

The title 'Turning the tide on ovarian cancer' is though a bit too catchy in my view.

**Authors’ response:**

*'Turning the tide on ovarian cancer' was removed from the title and wording rearranged as an alternative. We like to leave the decision on the title to the editorial team.*

The figures need revision: Figure 1 remained incomprehensible for me even after reading the legend multiple times.

***Re****: Figure 1 was re-designed in order to enhance comprehensibility.*

In figure 2, the y-axis is labeled with risk per year but I am unsure if you mean risk for surgery or risk for ovarian cancer.

**Authors’ response:**

*The meaning of risk per year is now clearly stated in the legend of figure 2.*

Figure 3 is a tough one as well as I could not read the y-axis nor understand the abbreviation WTP.

**Authors’ response:**

*Figure 3 was re-designed with larger font size and explanation of abbreviations is given in the legend. The willingness-to-pay threshold (WTP) is now abandoned in favor of 1x and 2x the gross domestic product per capita in Germany (2022) ^2^, which are often used as comparative values in the relevant literature.*

**Section 5: Response to Reviewer #4’s Comments**

**Reviewer #4:** Thank you to the authors for submitting this paper. I think the paper has the potential for publication in this journal, however there are several important changes I would make before publication. I have presented them in the order in which they appear in the paper.

Line 41: You report ICERs as your primary outcome measure. What relevance do they have in the German health care system? I'm aware that there is not an official threshold...

**Authors’ response:**

*Thank you for this important comment! The primary outcome of our study is clinical effectiveness, represented by prevented ovarian cancer cases and deaths. Because OS has already been implemented by gynecologic surgeons in several countries^3,4^ without clarified financial compensation, we also wanted to address this aspect by including the incremental cost-effectiveness ratio (ICER) as an economic outcome measure. Sometimes, the ICER (incremental cost-effectiveness ratio) is called ICUR (cost-utility ratio) if it refers to costs per QALY gained (calculated based on utility of a life year in a specific condition) instead of costs per life year gained. In order to avoid confusion, we now defined our primary outcome to be the cost-effectiveness ratio (ICER) expressed as costs per QALY and used it in this way throughout the manuscript.*

*In Germany, although there is no official ICER threshold, the ICER provides useful insights for decision-making bodies such as the Gemeinsamer Bundesausschuss (G-BA) and the Institut für Qualität und Wirtschaftlichkeit im Gesundheitswesen (IQWiG). These bodies consider cost-effectiveness data as part of their evaluations for new interventions, even if thresholds are not formally applied. The ICER is thus indirectly relevant as it informs reimbursement and resource allocation decisions, supporting value-based healthcare.*

*To enhance comparability with other international health systems, we have contextualized our ICER findings using Germany’s 2022 gross domestic product as a reference threshold in Figure 3 and in the text (p. 15, lines 376-381), which aligns with accepted practices in German health-economic evaluations.*

*Globally, ICER values are widely used by health authorities. The US Centers for Medicare & Medicaid Services and agencies in countries like Sweden and the UK, to our knowledge, use these measures to guide cost-effectiveness evaluations and reimbursement policies. In emerging markets such as India and China, ICER analyses are also becoming increasingly relevant for determining the economic value of healthcare interventions. This broader international perspective provides context for our model and allows comparisons with cost-effectiveness studies from other countries.*

Line 89: Could you please further justify your model structure? There seems to be a large number of health states? Other semi markov models I've seen in the literature have used relatively simple model structures (progression free, post-progression, death). Does it need to be that complicated?

**Authors’ response:**

*We have redesigned Figure 1 to improve clarity. The relatively high number of health states in our model is necessary to account for the different levels of ovarian cancer risk reduction associated with each intervention, as well as the possibility of transitions between these states. We carefully optimized the model structure to balance simplicity with the need to accurately reflect these distinctions. The structure of our model is consistent with similar studies ^1,5^ that analyze the effectiveness of prophylactic or opportunistic salpingectomy.*

Line 120: What is the evidence on which the assumption that mortality returns back to background after 10 years based on?

**Authors’ response:**

*We admit that this is a simplified assumption. However, no data an actual mortality >10 years after diagnosis are available and ovarian cancer mortality in our model closely reproduces real-world data. We now indicated on p 8 lines 194-195 that it is an assumption.*

Line 137-Line 140: The justification for these parameters seems a little suspect. Please further expand on the justification.

**Authors’ response:**

The adjustment and alignment with the observed rate were necessary because most tubal sterilization procedures are performed in an outpatient setting and are not covered by the health insurance. Case numbers for such outpatient self-payed surgeries are currently not recorded in Germany. This is now explicitly stated on p. 9, lines 212-219. In order to explore the impact of the used calibration and varying information regarding the observed rate on the results, different transition probabilities for sterilization were examined during sensitivity analysis (methods section p. 11, lines 284-287; results section p. 14 line 347; supplementary tables S15 and S16).

Line 150: A utility value of 1 should not be used for healthy states. The age-adjusted population average should be used. This is common practice in the literature.

**Authors’ response:**

*Thank you very much for this suggestion. We now ran the analysis of our model with age-adjusted population average utilities for healthy states^6^. This is now indicated on p. 9 line 229 and numbers were accordingly corrected in the results section as well as in table 1 and figure 3.*

Line 158: Please provide further evidence that the costs of the interventions should not be included.

**Authors’ response:**

*Thank you for this important question. We assume that surgeries with the opportunity for OS are performed at the same frequency across all four analyzed strategies. Currently, it is not recommended to perform surgery solely for the purpose of risk reducing salpingectomy. Instead, salpingectomy is only performed opportunistically during a medically necessary gynecological or non-gynecological abdominal intervention or if a woman chooses permanent contraception. Consequently, the costs of these primary surgeries are independent of the decision to perform OS and were therefore not included in the analysis.*

*To clarify this, we inserted a new subsection “Basic considerations and assumptions” at the beginning of the methods section (p. 7 lines 145-151), containing the following statement: “For women with average cancer risk, it is currently not recommended to perform a surgery only because OS is desired. OS is thought to be performed exclusively on occasion of a gynecological or non-gynecological abdominal intervention that is medically necessary, or if a woman decides to have permanent contraception. Consequently, the number and type of surgeries with opportunity for OS is the same in all strategies analyzed in this study. Therefore, costs and risks of these surgeries were not considered.”*

Line 179: Please provide further justification that a one year cycle length is appropriate in this instance. What is this based on? Previous models in this area I've seen have used monthly cycles.

**Authors’ response:**

*Our model represents a lifetime analysis and the time horizon for occurrence of events is years, not months. This becomes obvious from the intervals between relevant events. For example, the average age for hysterectomy (with opportunity for OS) is 44 years^7^, while the average age for onset of OC is 69 years^8^, resulting in an interval of more than 20 years. Furthermore, publications with similar models on opportunistic or prophylactic salpingectomy also used a cycle length of one year.^1,5^ This is now also indicated in the revised manuscript on page 10 line 260.*

Line 179: Was half cycle correction used? If not, why not?

**Authors’ response:**

*Yes, half-cycle correction was used. This is now stated in the revised manuscript at p. 10 lines 257-260.*

Line 188: Please define what you mean by "stable".

**Authors’ response:**

*Because of low ovarian cancer incidence, the model yielded variable results for OC incidence, mortality, as well as ICUR and variance was high in analysis with < 1,200,000 individuals. This is now shown in new supplemental figures S12-S15.*

Line 192: Transition probabilities should be included in the sensitivity analysis, it doesn't matter if they are based on real-world population data or not.

**Authors’ response:**

*Because the transition probabilities are calculated from real-world population level data, they do not contain the problems associated with parameter estimation (parameter uncertainty) and heterogeneity between individuals ^9^. However, from a scenario analysis view, it could have an impact if incidence of surgeries with opportunity for OS would change. For example, the number of hysterectomies significantly declined during the past 15 years due to the trend to organ preserving therapy^10^. This is in part, but not fully, compensated by the implementation of myomectomy into our model. To explore this in more detail, we performed DSA with 50% incidence of hysterectomy. Furthermore, it is conceivable that recent improvements in OC treatment (introduction of maintenance therapy) could lead to an improved prognosis. To reflect this, DSA was performed with reduced risk for death after OC diagnosis. These considerations are now included into the Methods section on p. 11 lines 274-287. Results are shown in supplemental tables S15 and S16 and in the main text body on p. 14 line 347.*

Line 211: Please comment on why the prevalence may be lower in the model compared to the published data.

**Authors’ response:**

*To explain this in more detail, we revised and expanded the paragraph on p. 12 lines 305-310 to give the following wording: “The prevalence of 14% for hysterectomy in women at age 70 years or older in our model is considerably lower compared to 39.4% found in a survey conducted in Germany during years 2008-11^7^. However, all hysterectomies were counted in the publication by Prütz et al., while we only included hysterectomies for benign indications. Furthermore, hysterectomy case numbers almost halved since the beginning of the millennium^10^, resulting in lower prevalence in the long term.”*

Table 1: Is is possible to use a consistent number of decimal places for the various parameters/results?

**Authors’ response:**

*Of course. We corrected all values accordingly. We decided to consistently use two decimal places for percentages and costs and five decimal places for life years and QALYs per capita.*

Line 273: A probabilistic sensitivity analysis needs to be included in analyses such as this in order to fully explore the uncertainty in the model.

**Authors’ response:**

*In the deterministic sensitivity analysis, critical impact was only seen for ovarian cancer risk reduction after salpingectomy. In the revised version of the manuscript, a probabilistic sensitivity analyses is therefore conducted for variations in risk reduction in combination with a variation of costs of OS. Results are presented in the main text body p. 15 lines 383-389 and supplemental figure S18.*

Line 350: How important is the lack of discrimination between sub types of ovarian cancer? How would the inclusion of these sub-types impact the results?

**Authors’ response:**

*Thank you for this insightful question. To clarify the issue of varying risk reduction for different histologic subtypes (ex.g. high grade serous or endometroid vs. ex.g. mucinous), we have added the following statement in a new paragraph titled “Basic Considerations and Assumptions” at the beginning of the Methods section:*

*“Ovarian cancer risk reduction after removal of fallopian tubes might be different for histologic subtypes, due to the divers origin of precursor lesions. Furthermore, there are women with increased ovarian cancer risk, e.g. BRCA (BReast CAncer gene) mutation carriers. However, all numbers used to inform the model do not discriminate between histological subtypes and between women with different cancer risk. Consequently, the results of the model represent estimates for the impact of OS on overall ovarian cancer incidence, mortality and healthcare costs on a population level. Women with increased cancer risk need special consultation and are recommended to have bilateral salpingo-oophorectomy between 35 and 45 years of age.” (p. 7 lines 152-160). The confusing sentence in the limitations section was removed (p. 19 line 486).*

I will comment further on the results in the next round of peer review once a PSA has been provided.

**Section 6: Additional modifications**

- The name of one of the authors was corrected (Habib Arefian instead of Habibollah Arefian)
- Correction of wording on
  - p. 6, lines 140-144
  - p. 16 line 409-411: Insertion of the sentence: “In consideration of the fact that ovarian cancer still is a burdensome and deadly disease and no effective early diagnosis strategy is available, prevention of approximately 10% of cases per year would be a great achievement.
  - Table 1 (Carcinoma > cancer)
- The following sentences were included on p. 14 lines 350-352 in order to emphasize importance of OS: *“In consideration of the fact that ovarian cancer still is a burdensome and deadly disease and no effective early diagnosis strategy is available, prevention of approximately 700 cases per year would be a great achievement.”*
- The following sentences were included on p. 16 lines 414-518 in order to further expand on health-economic aspects: *“Our data indicate, that reimbursement of OS up to 332 € could be possible without additional expenditures for the healthcare system. Partial cost coverage for out-patient sterilization procedures, which currently is a self-payer service, would be desirable and could be justified because of efficient cancer prevention.”*
- The following sentences were included on page 17 lines 446-451 in order to discuss in more detail the aspect of patient counselling and attitudes*: “Use of a structured and validated decision aid^11^ might help to standardize counselling and ensure quality. Some studies with a small number of participants indicate that the majority of women would opt for OS as the permanent contraception method^12–14^ after appropriate counselling, because of the presumably lower failure rate and the expected reduction in risk for ovarian cancer.”*
- The following sentences were included on page 17 lines 427-430 in order to touch on possible increase in tubal sterilization incidence: *“However, in recent years, there has been an increased demand for tubal sterilization in several countries, possibly due to concerns over a slightly increased risk of breast cancer associated with hormonal contraception.”*
- The following sentences were included on page 17 lines 439-443 in order to reference a recent study on possible complications after OS: *“A Swedish national register-based randomized non-inferiority trial on salpingectomy, the SALSTER study, has just recently demonstrated as its main result that laparoscopic salpingectomy does not increase the risk of complications compared to tubal occlusion up to eight weeks postoperatively, confirming it as a safe alternative^15^.“*

**Section 7: Response to general editorial requests:**

*	We ask every co-author listed on the manuscript to fill in a contributing author statement, making sure to declare all competing interests. If any of the co-authors have not filled in the statement, we will remind them to do so when the paper is revised. If all statements are not completed in a timely fashion this could hold up the re-review process. If new competing interests are declared later in the revision process, this may also hold up the submission. Should there be a problem getting one of your co-authors to fill in a statement we will be in contact. Please do not add or remove authors without first discussing this with the handling editor. You can see our competing interests policy here: <http://journals.plos.org/plosmedicine/s/competing-interests>.

*Authors response:*

*Signed COI Statements were obtained from all authors and uploaded.*

*	Please upload any figures associated with your paper as individual TIF or EPS files with 300dpi resolution at resubmission; please read our figure guidelines for more information on our requirements: <http://journals.plos.org/plosmedicine/s/figures>. While revising your submission, please upload your figure files to the PACE digital diagnostic tool, <https://pacev2.apexcovantage.com/>. PACE helps ensure that figures meet PLOS requirements. To use PACE, you must first register as a user. Then, login and navigate to the UPLOAD tab, where you will find detailed instructions on how to use the tool. If you encounter any issues or have any questions when using PACE, please email us at [PLOSMedicine@plos.org](mailto:PLOSMedicine@plos.org).

*Authors response:*

*Figures were prepared accordingly and checked with PACE before uploading.*

*	Please ensure that the paper adheres to the PLOS Data Availability Policy (see <http://journals.plos.org/plosmedicine/s/data-availability>), which requires that all data underlying the study's findings be provided in a repository or as Supporting Information. For data residing with a third party, authors are required to provide instructions with contact information (web or email address) for obtaining the data. Please note that a study author cannot be the contact person for the data. PLOS journals do not allow statements supported by "data not shown" or "unpublished results." For such statements, authors must provide supporting data or cite public sources that include it.

*Authors response:*

*All data is included in the manuscript or in the submitted supporting information file.*

*	We expect all researchers with submissions to PLOS in which author-generated code underpins the findings in the manuscript to make all author-generated code available without restrictions upon publication of the work. In cases where code is central to the manuscript, we may require the code to be made available as a condition of publication. Authors are responsible for ensuring that the code is reusable and well documented. Please make any custom code available, either as part of your data deposition or as a supplementary file. Please add a sentence to your data availability statement regarding any code used in the study, e.g. "The code used in the analysis is available from Github [URL] and archived in Zenodo [DOI link]" Please review our guidelines at <https://journals.plos.org/plosmedicine/s/materials-software-and-code-sharing> and ensure that your code is shared in a way that follows best practice and facilitates reproducibility and reuse. Because Github depositions can be readily changed or deleted, we encourage you to make a permanent DOI'd copy (e.g. in Zenodo) and provide the URL.

*Authors response: Not applicable.*

FORMATTING - GENERAL

*	Abstract: Please structure your abstract using the PLOS Medicine headings (Background, Methods and Findings, Conclusions). Please combine the Methods and Findings sections into one section.

*Authors response:*

*Abstract was structured accordingly.*

*	At this stage, we ask that you include a short, non-technical Author Summary of your research to make findings accessible to a wide audience that includes both scientists and non-scientists. The Author Summary should immediately follow the Abstract in your revised manuscript. This text is subject to editorial change and should be distinct from the scientific abstract. Ideally each sub-heading should contain 2-3 single sentence, concise bullet points containing the most salient points from your study. In the final bullet point of 'What Do These Findings Mean?', please include the main limitations of the study in non-technical language. Please see our author guidelines for more information: <https://journals.plos.org/plosmedicine/s/revising-your-manuscript#loc-author-summary>.

*Authors response:*

*The non-technical author summary was now included.*

*	Please express the main results with 95% CIs as well as p values. When reporting p values please report as p<0.001 and where higher as the exact p value p=0.002, for example. Throughout, suggest reporting statistical information as follows to improve clarity for the reader "22% (95% CI [13%,28%]; p</=)". Please be sure to define all numerical values at first use.

*Authors response: Not applicable.*

*	Please include page numbers and line numbers in the manuscript file. Use continuous line numbers (do not restart the numbering on each page).

*Authors response: Done.*

*	Please cite the reference numbers in square brackets. Citations should precede punctuation.

*Authors response: Done.*

FIGURES AND TABLES

*	Please provide titles and legends for all figures and tables (including those in Supporting Information files).

*Authors response: Done.*

*	Please define all abbreviations used in each figure/table (including those in Supporting Information files).

*Authors response: Done.*

*	Please consider avoiding the use of red and green in order to make your figure more accessible to those with color blindness.

*Authors response:*

*Figures were revised to adhere to this request.*

SUPPLEMENTARY MATERIAL

*	Please note that supplementary material will be posted as supplied by the authors. Therefore, please amend it according to the relevant comments outlined here.

*	Please cite your Supporting Information as outlined here: <https://journals.plos.org/plosmedicine/s/supporting-information>

*Authors response:*

Supplementary tables and figures were prepared as requested and cited at the appropriate location in the main text body.

REFERENCES

*	PLOS uses the numbered citation (citation-sequence) method and first six authors, et al.

*	Please ensure that journal name abbreviations match those found in the National Center for Biotechnology Information (NCBI) databases (<http://www.ncbi.nlm.nih.gov/nlmcatalog/journals>), and are appropriately formatted and capitalised.

*	Where website addresses are cited, please include the complete URL and specify the date of access (e.g. [accessed: 12/06/2024]).

*	Please also see <https://journals.plos.org/plosmedicine/s/submission-guidelines#loc-references> for further details on reference formatting.

*Authors response: References are cited according to PLOS Medicine requirements.*

STUDY TYPE-SPECIFIC REQUESTS

The following list is derived from Geoffrey P Garnett, Simon Cousens, Timothy B Hallett, Richard Steketee, Neff Walker. Mathematical models in the evaluation of health programmes. (2011) Lancet DOI:10.1016/S0140-6736(10)61505-X:

*	If pertinent, please provide a diagram that shows the model structure, including how the natural history of the disease is represented, the process and determinants of disease acquisition, and how the putative intervention could affect the system.

*Authors response: A diagram of the model is depicted in Figure 1.*

*	Please provide a complete list of model parameters, including clear and precise descriptions of the meaning of each parameter, together with the values or ranges for each, with justification or the primary source cited and important caveats about the use of these values noted.

*Authors response: Done. (Methods section and supplementary material Tables S1-S11, S17)*

*	Please provide a clear statement about how the model was fitted to the data, including goodness-of-fit measure, the numerical algorithm used, which parameter varied, constraints imposed on parameter values, and starting conditions.

*Authors response: Not applicable.*

*	For uncertainty analyses, please state the sources of uncertainties quantified and not quantified [can include parameter, data, and model structure].

*Authors response: Done. (Methods section)*

*	Please provide sensitivity analyses to identify which parameter values are most important in the model. Uncertainty estimates seek to derive a range of credible results on the basis of an exploration of the range of reasonable parameter values. The choice of method should be presented and justified.

*Authors response: Done.*

*	Please discuss the scientific rationale for the choice of model structure and identify points where this choice could influence conclusions drawn. Please also describe the strength of the scientific basis underlying the key model assumptions.

*Authors response: Done.*

*	For studies that develop a prediction model or evaluate its performance, please ensure that the study is reported according to the TRIPOD statement (<https://www.equator-network.org/reporting-guidelines/tripod-statement>) and include the completed checklist as Supporting Information. Please add the following statement, or similar, to the Methods: "This study is reported as per the Transparent Reporting of a Multivariable Prediction Model for Individual Prognosis Or Diagnosis (TRIPOD) statement (S1 Checklist)." For studies using machine learning, please use the TRIPOD-AI checklist. When completing the checklist, please use section and paragraph numbers, rather than page numbers.

*Authors response: Not applicable.*

*	For the cost-effectiveness section of your manuscript, please consider the items outlined in the CHEERS guideline/checklist (available at <https://www.equator-network.org/reporting-guidelines/cheers>).

*Authors response: Done.*

Sincerely yours, for the authors

Ingo B. Runnebaum, MD, Dr. h.c., MBA	Angela Kather, PhD, MSc

Corresp. author for gynecology	Corresp. author for med. informatics

----------------------------------------------

Professor of Gynecology
State Medical Association Recognitions:
(1) Specialist in Obstetrics and Gynecology
(2) Subspecialty in Gynecologic Oncology
(3) Subspecialty in Gynecologic Endocrinology and Reproductive Medicine
(4) Subspecialty in Specialized Obstetrics and Perinatal Medicine
(5) Qualification in Specialized Surgical Gynecology

Recognitions from other Societies:
MIC III (highest level, Minimal-Invasive Surgery, certified by DGGG/AGE)
Senior-Breast Surgeon (Deutsche Krebsgesellschaft/Onkozert)

References

1.	Hallsson LR, Sroczynski G, Engel J, et al. Decision-analytic evaluation of the comparative effectiveness and cost-effectiveness of strategies to prevent breast and ovarian cancer in German women with BRCA-1/2 mutations. BMC Cancer 2023; 23: 590.

2.	Statistisches Bundesamt. Volkswirtschaftliche Gesamtrechnungen - Wichtige Zusammenhänge im Überblick – Stand: August 2024 2024.

3.	Ntoumanoglou-Schuiki A, Tomasch G, Laky R, et al. Opportunistic prophylactic salpingectomy for prevention of ovarian cancer: What do national societies advise? Eur J Obstet Gynecol Reprod Biol 2018; 225: 110–112.

4.	Runnebaum IB, Kather A, Vorwergk J, et al. Ovarian cancer prevention by opportunistic salpingectomy is a new de facto standard in Germany. J Cancer Res Clin Oncol 2023; 149: 6953–6966.

5.	Naumann RW, Hughes BN, Brown J, et al. The impact of opportunistic salpingectomy on ovarian cancer mortality and healthcare costs: a call for universal insurance coverage. Am J Obstet Gynecol 2021; 225: 397.e1-397.e6.

6.	Grochtdreis T, Dams J, König H-H, et al. Health-related quality of life measured with the EQ-5D-5L: estimation of normative index values based on a representative German population sample and value set. Eur J Health Econ 2019; 20: 933–944.

7.	Prütz F, Knopf H, Lippe E von der, et al. Prevalence of hysterectomy in women 18 to 79 years old: results of the German Health Interview and Examination Survey for Adults (DEGS1). Bundesgesundheitsblatt Gesundheitsforschung Gesundheitsschutz 2013; 56: 716–722.

8.	Buttmann-Schweiger N and Kraywinkel K. Epidemiologie von Eierstockkrebs in Deutschland. Onkologe 2019; 25: 92–98.

9.	Briggs AH, Weinstein MC, Fenwick EAL, et al. Model parameter estimation and uncertainty analysis: a report of the ISPOR-SMDM Modeling Good Research Practices Task Force Working Group-6. Med Decis Making 2012; 32: 722–732.

10.	Runnebaum IB, Kather A and Sehouli J. Opportunistic Salpingectomy for the Primary Prevention of Ovarian Cancer. Dtsch Arztebl Int 2022; 119: 846–847.

11.	van Lieshout LAM, Gelderblom ME, Hullu JA de, et al. Primary prevention of ovarian cancer: a patient decision aid for opportunistic salpingectomy. Am J Obstet Gynecol 2022; 226: 234.e1-234.e14.

12.	Zerden ML, Castellano T, Doll KM, et al. Risk-Reducing Salpingectomy Versus Standard Tubal Sterilization: Lessons From Offering Women Options for Interval Sterilization. South Med J 2018; 111: 173–177.

13.	Tomasch G, Bliem B, Lemmerer M, et al. Would women accept opportunistic (prophylactic) salpingectomy at the time of nongynecologic surgery to prevent development of ovarian cancer? Surgery 2018; 164: 931–934.

14.	Piazza A, Schwirian K, Scott F, et al. Women's preferences for permanent contraception method and willingness to be randomized for a hypothetical trial. Contraception 2019; 99: 56–60.

15.	Strandell A, Magarakis L, Sundfeldt K, et al. Salpingectomy versus tubal occlusion in laparoscopic sterilisation (SALSTER): a national register-based randomised non-inferiority trial. The Lancet Regional Health - Europe 2024; 45: 101026.
